# Surgical Correction of Kyphosis in Patients With Camptocormia Associated With Parkinson’s Disease: A Case Report and Review of the Literature

**DOI:** 10.3389/fsurg.2022.822015

**Published:** 2022-06-29

**Authors:** Guo-long Mei, Hui-ting Wei, Yue-rong Ma, Dun Wan

**Affiliations:** ^1^ Spine Department, Sichuan Province Orthopedic Hospital, Chengdu, China; ^2^ School of Basic Medicine, Chengdu University of Traditional Chinese Medicine, Chengdu, China

**Keywords:** camptocormia, Parkinson, revision surgery, surgical correction of kyphosis, treatment decision

## Abstract

**Background:**

Camptocormia is a postural deformity that is characterized by a markedly flexed lumbar spine, with symptoms that worsen with walking and standing. Here, we report a case of camptocormia associated with Parkinson’s disease.

**Case description:**

A 70-year-old man with a 7-year history of Parkinson’s disease presented with a fall injury that caused lower back pain for 3 months and was aggravated for 2 months. He had been diagnosed with a compression fracture after the fall and had undergone percutaneous kyphoplasty at a local hospital. MRI showed non-union of the L1 vertebra and compression fracture of L2. The patient underwent posterior osteotomy, canal decompression, and internal fixation of the T10-L3 intervertebral plate with bone graft fusion. Postoperative examination showed that the lumbar lordosis was corrected and sensation was restored in both lower extremities. However, after 1 month, the fixation was loosened and a correction surgery was performed at our hospital. At the most recent follow-up at 1.5 years, the patient was found to be in good general health and did not complain of lower back discomfort. He was also actively exercising according to the rehabilitation regimen and had resumed social life.

**Conclusion:**

This is a rare case of camptocormia in a Parkinson’s patient that highlights the need for careful evaluation of whether internal spinal fixation surgery is beneficial in such patients.

## Introduction

Camptocormia is a kyphotic deformity that is characterized by a markedly flexed thoracic cage and lumbar spine, with symptoms that worsen with walking and standing and decrease with lying down. The term “camptocormia” was coined by two French neurologists by combining the Greek words *kamptos*, which means “curved,” and *kormos*, which means “trunk.” The term was used by Brodie as early as 1837 to describe the exaggerated bending of the spine. The etiology of camptocormia is complex, and Parkinson’s disease is one of the most important causes. A single-center study showed that the incidence of camptocormia in patients with Parkinson’s disease is 6.9% (1), and another study showed that 11 of 16 patients with Parkinson’s had camptocormia (2). Further, a study of 1453 Parkinson’s patients by Japanese scholars showed that 9.5% of the patients developed camptocormia 8.1 years after onset (3). Additionally, it was more common among female patients than among male patients, and patients with late-onset Parkinson’s were more common than those with early-onset Parkinson’s (3). It has been found that the signs of camptocormia occur sequentially after the onset of Parkinson’s symptoms, and that the incidence of camptocormia is positively correlated with the severity of Parkinson’s symptoms (1). Thus, although camptocormia is an uncommon condition, Parkinson’s disease and camptocormia seem to be closely related.

The available treatments for camptocormia include oral and intramuscular drugs and surgical treatments such as deep brain stimulation and orthopedic spinal surgery. However, in a retrospective study of 12 patients with Parkinson’s who developed camptocormia, no significant symptom relief was found with oral levodopa treatment. Moreover, in another group of nine people who were treated with botulinum toxin type A via rectus abdominis injection, only four experienced symptom relief. Further, no symptom relief was reported with bilateral deep subthalamic nucleus stimulation in one case (2). In a study of 34 patients, discontinuation of pramipexole for Parkinson’s was found to alleviate camptocormia symptoms in some patients. Investigators believe that it is probably because Parkinson’s is an axial dystonia rather than a myelopathy. However, whether camptocormia associated with Parkinson’s can benefit from discontinuing pramipexole is something that needs to be further evaluated (3). With regard to the surgical modalities, a meta-analysis has suggested that, although the use of deep stimulation improved the sagittal imbalance of the spine by 50% in 36.4% of patients, its treatment effect was inconsistent (4). Further, orthopedic spine surgery can completely correct the spinal imbalance, but it is associated with several complications (4). In general, the current studies imply that the efficacy of the treatment modalities for camptocormia is unclear because the etiologies are diverse.

In this article, we report the treatment of a patient with camptocormia caused by Parkinson’s disease. Based on the findings, we emphasize on the need for careful assessment of whether this group of patients can benefit from surgery through a review of the medical history and analysis of the current data.

## Case Presentation

The patient was a 70-year-old man with a medical history of Parkinson’s disease for 7 years and good disease control through regular treatment with the oral medications levodopa and pramipexole. The patient had undergone rectal polypectomy 1 year ago, but he did not have a remarkable medical history otherwise. He did not have a history of alcohol consumption or smoking. His chief complaint was a 3-month history of pain in the lower back after falling down that had been exacerbated for 2 months.

**Figure 1:**
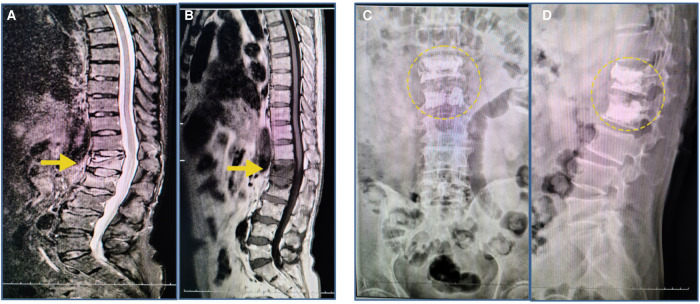
MRI findings after the initial fall; radiographs after the PKP surgery. (**A**,**B**) T1-weighted images revealed compression fractures of the L1 and L2 vertebrae (yellow arrow). (**C**,**D**) The radiograph image reveals L1 and L2 vertebrae that were treated with percutaneous kyphoplasty (yellow circle).

**Figure 2:**
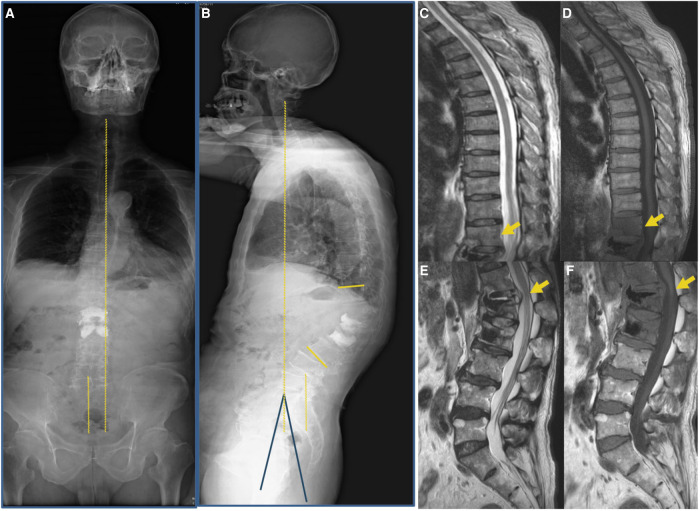
Images were taken at the initial visit to our hospital. (**A**,**B**) A full-length radiograph of the patient’s spine indicated sagittal imbalance of the spine (Cobb’s angle: 45°, PI: 37°, PT: 26°, SS: 11°). (**C**–**F**) MRI indicated post-vertebroplasty of L1 and L2 and T12-L2 vertebral body with bone edema (yellow arrow).

Three months back, the patient fell off the bed and experienced mild back pain. He was admitted to the local hospital, where he was diagnosed with vertebral compression fractures of L1 and L2 and osteoporosis (Figures 1A,B). He then underwent percutaneous kyphoplasty, after which the pain was mildly relieved (Figures 1C,D). Two months after the kyphoplasty procedure, his back pain gradually worsened and he also started experiencing radiating pain in the left inguinal and left lateral thigh. He was unable to sit, stand, and walk as a result of the increasing pain, and also experienced weakness while defecating. Physical examination revealed deformity of the thoracolumbar segment in the form of a posterior convexity, as well as localized pressure and percussion pain in the thoracolumbar segment, slight tension in the paravertebral muscles, and dullness of sensation in the left inguinal and lateral thigh. No pathologic signs were observed, but lumbar spine motion was limited. A full-length spine radiograph showed post-vertebroplasty of L1 and L2, compression fracture of T12, kyphosis with sagittal imbalance (Cobb’s angle: 45°, PI: 37°, PT: 26°, SS: 11°), and severe osteoporosis (Figures 2A,B). MRI of the lumbar spine showed T12-L2 bone edema (Figures 2C,F). Laboratory examination showed that the erythrocyte sedimentation rate (ESR) increased to 54.00 mm/h and hemoglobin (HGB) decreased to 113 g/L.

### Initial Surgery at Our Hospital

**Figure 3:**
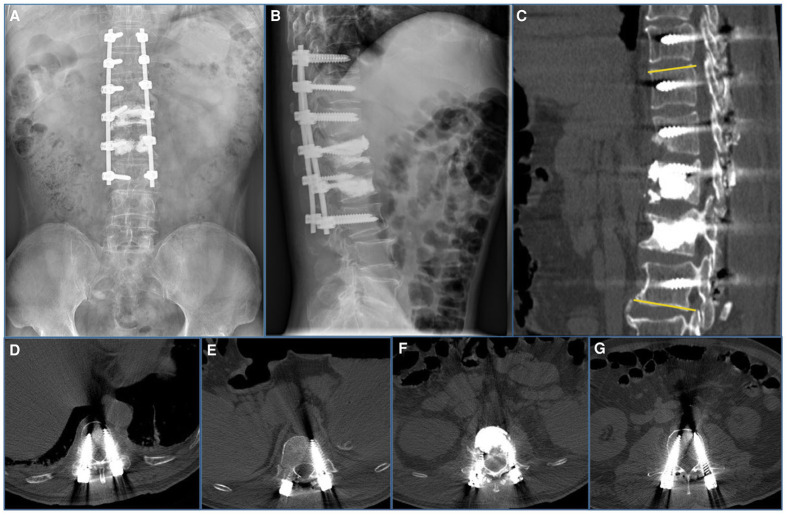
Imaging features after initial surgery at our hospital. (**A**,**B**) Postoperative 1-week radiograph indicated alleviation of the kyphosis. (**C**–**G**) Postoperative 1-week CT scan indicated good internal screw fixation (Cobb’s angle: 10°).

Based on the observations, imaging findings, and the patient’s needs, posterior osteotomy, canal decompression, and internal fixation of the T10-L3 intervertebral plate with bone graft fusion was selected as the treatment option. After the surgery, the patient got out of bed with the protection of a brace, and the pain in the lower back was reduced. Additionally, sensation in the lower limbs was normal, and bowel movement was normal too. The surgical incision healed well and met the criteria for first-stage healing. Postoperative lumbar spine radiography and CT showed that the kyphosis was corrected and the nail rod was not loosened (Figures 3A–F). The patient was satisfied with the outcome of the surgery and was discharged from the hospital. He was asked to continue with oral Parkinson’s therapy with additional bisphosphonates, calcium, and vitamin D3 for anti-osteoporosis treatment. The patient was instructed to wear a brace during all daily activities.

**Figure 4:**
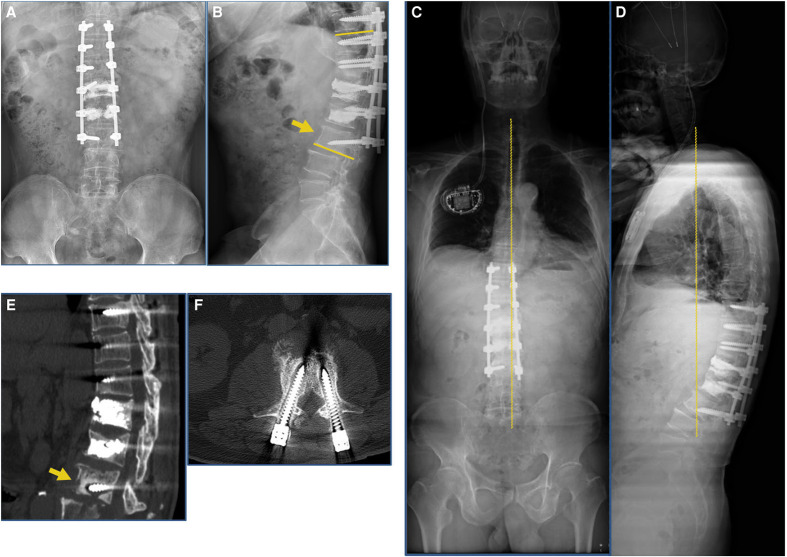
Postoperative 1-month and 3-month imaging findings at our hospital. (**A**,**B**) Postoperative 1-month radiograph indicated loosening of the internal fixation screw of the L3 vertebral body (yellow arrow). (**C**,**D**) Postoperative 3-month follow-up full-length radiograph of the patient’s spine indicated Cobb’s angle increased to 26°, failure of internal fixation. (**E**,**F**) Postoperative 3-month follow-up CT scan indicated that the L3 vertebral body was loosely fixed internally and the screws had resorbed the surrounding bone (yellow arrow).

Unfortunately, a follow-up radiograph (Figures 4A,B) taken 1 month after surgery showed a loosening of the L3 pedicle screw. The patient was advised to continue anti-osteoporosis treatment and Parkinson’s treatment and wear a brace and was counseled in preparation for possible secondary surgery.

### Revision Surgery at Our Hospital

Three months after surgery, the patient complained of lower back pain that worsened when walking upright and turning over. Physical examination revealed that in-surgical incision was well healed, there was localized pressure and percussion pain in the distal fixed vertebrae segment, and the muscle strength of the lower limbs was normal. No pathologic signs were observed. CT scan and radiograph of the spine showed that the L3 vertebral body was loosely fixed internally and the screws had resorbed the surrounding bone (Figures 4C–F).

**Figure 5:**
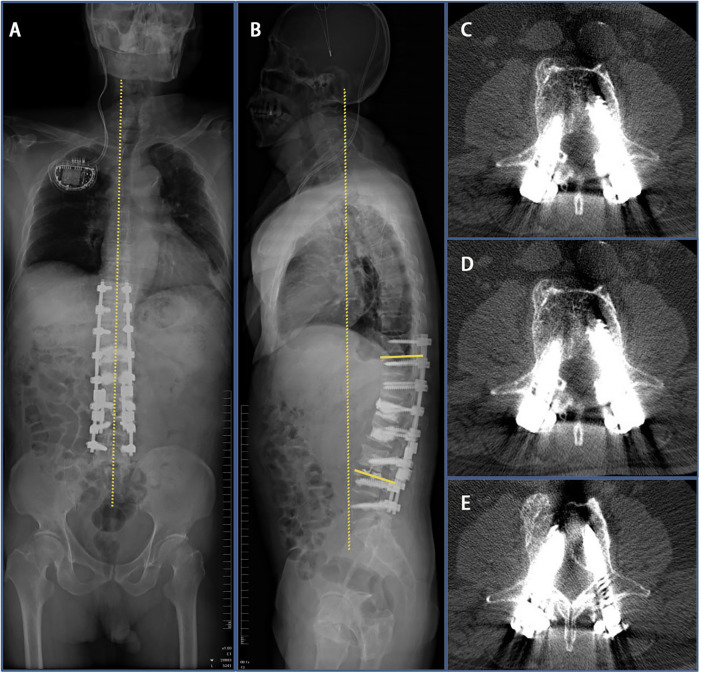
Imaging findings 3 days after revision surgery at our hospital. (**A**,**B**) 3 days after revision surgery full-length radiograph of the patient’s spine indicated that Cobb’s angle decreased to 20°. (**C**–**E**) CT scan indicated good internal screw fixation.

Two months after initial surgery, the patient underwent deep-brain electrode placement at West China Hospital. Based on the symptoms, imaging findings, and the patient’s needs, the revision surgery was chosen to be performed. As the original internal fixation had failed and the patient had severe osteoporosis, the original L3 screw used for internal fixation was removed. Additionally, the L3–5 vertebral body was lengthened and fixed, the L3–5 vertebral nail tract was reinforced with bone cement, and a Domino joint head device was installed. After revision surgery, physical examination showed that the local pressure pain and percussion pain had been alleviated. Imaging 3 days after revision surgery showed that kyphosis was corrected (Cobb’s angle: 22°, PI: 44°, PT: 23°, SS: 21°) and the nail rod was not loosened (Figures 5A–E). At the time of discharge, the patient was instructed to continue wearing the brace and continue with active anti-osteoporosis treatment, with regular review of his condition.

**Figure 6:**
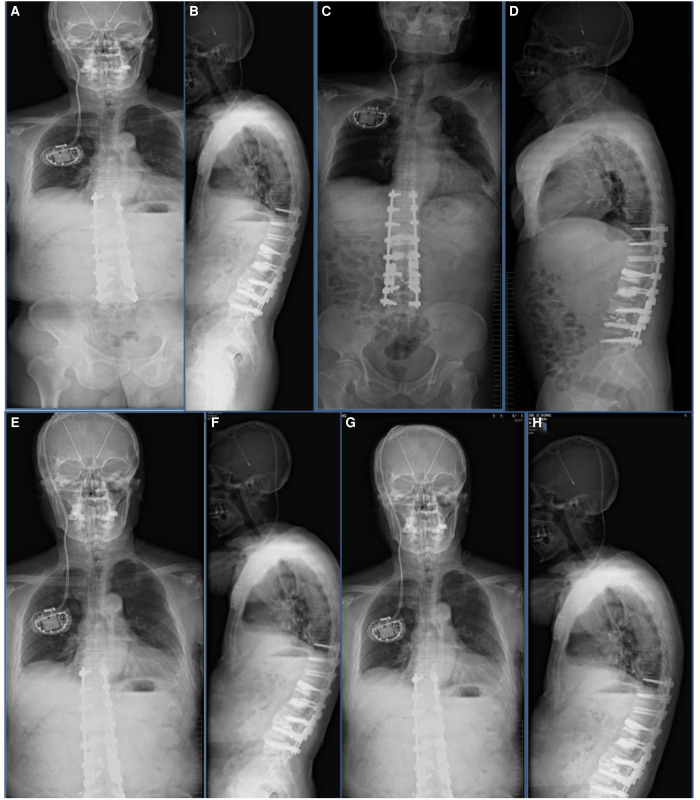
Imaging findings after revision surgery at our hospital. (**A**,**B**) One-month follow-up postoperative radiograph indicated good spinal stability without loosening of the screws. (**C**,**D**) Three-month follow-up postoperative radiograph indicated good spinal stability without loosening of the screws. (**E**,**F**) The patient’s radiograph was reviewed 6 months after surgery and showed the good orthopedic status of the spine without loosening of the internal fixation screws. (**G**,**H**) Follow-up radiographs taken 1.5 years after surgery showed no loosening of the internal fixation and good spinal orthopedic status.

One month after revision surgery, imaging showed that the kyphosis was corrected and the internal fixation was stable (Figures 6A,B). Two months after revision surgery, the brace was successfully removed. At 3-month and 6-month follow-ups, the patient was in good condition and the internal fixation was stable (Figures 6C–F). At the last follow-up conducted 1.5 years after the revision surgery (Figures 6G–H), the patient was in good condition and did not complain of lower back discomfort. He had been actively exercising according to the rehabilitation regime and had resumed social life.

## Discussion

Here, we report a case of camptocormia in a 70-year-old man with Parkinson’s disease who underwent spine surgery with posterior osteotomy, canal decompression, and internal fixation of the problematic intervertebral plate with bone graft fusion. Two other cases were reported in the literature, in which patients with Parkinson’s who developed camptocormia underwent orthopedic spine treatment: in one case, the patient was a 69-year-old man, and in the second case, the patient was a 77-year-old man (5). The 69-year-old patient developed lower back pain and radiating pain in his left thigh five years after the onset of Parkinson’s, and these symptoms were followed by postural disorders that forced him to give up his exercise routine. He also had several falls, was unable to live independently, and used a wheelchair to move around. Although the onset of camptocormia occurred a couple of years later in the present case, some of the symptoms were similar, for example, lower back pain and radiating pain in the lower extremities. In the second reported case in the literature, the 77-year-old patient had a 5-year history of Parkinson’s disease and a history of lumbar compression fracture. Similar to the second case, the patient in our case also had a history of lumbar compression fractures. Based on the symptoms in these reported cases, lower back pain and kyphotic deformity of the thoracolumbar region could be considered only as signs of camptocormia in patients with Parkinson’s.

Camptocormia is usually surgically treated with transient external spinal stimulation, deep brain stimulation, and orthopedic spine surgery (5–9). In the previous case of the 69-year-old patient, after thoroughly evaluating the patient’s condition and ruling out other causes of camptocormia, the surgeon recommended surgery. At the 5-year follow-up, the patient continued to use a walker for ambulation and his posture and gait had deteriorated, but he was satisfied with the results of the surgery because he was not dependent on a wheelchair. In the present case, the internal fixation had failed after the initial surgery in our hospital and had to be corrected by lengthening the fixation. In the case of the 77-year-old patient reported in the literature, only the first stage of surgery was performed without osteotomy on account of the poor general condition of the patient. However, left rod fracture occurred 24 months after surgery; the fracture may be related to mechanical stress caused by the absence of osteotomy. At follow-up, it was found that while his gait had initially improved after surgery, it gradually deteriorated over time to the same level as that before surgery. In comparison, in the present case, the patient was treated with vertebroplasty after the fall and was generally in good condition. However, the later complications probably occurred because the vertebral compression fractures that resulted from the fall were not optimally treated using vertebroplasty. At the time of writing this article, the patient had been followed up for 2 years and was recovering well. He was able to take care of himself and perform his daily exercises successfully. The patient was satisfied with the outcome of the treatment. Based on the findings in these three cases, surgical treatment might not always be appropriate in these patients due to their poor muscle strength and the high likelihood of the need for secondary revision surgery after internal fixation. Therefore, we recommend that patients with Parkinson’s who develop camptocormia should be carefully considered for surgery and do not recommend surgery as a first option.

Although the patient in the present case seems to be benefiting from the surgical treatment, a longer follow-up period is needed to understand the long-term benefits of surgical treatment. Parkinson’s associated with camptocormia is uncommon, but it severely affects the life of patients, some of whom have significant problems with activities of daily living. Importantly, there are no target treatment options, so the treatment options need to be carefully evaluated, especially the use of orthopedic spinal surgery.

## Conclusion

This is a rare case of camptocormia in a Parkinson’s patient that highlights the need for careful evaluation of whether internal spinal fixation surgery is beneficial in such patients.

## Data Availability

The data are available from the corresponding author upon request.
